# Consumption and Dogma

**Published:** 1910-02-19

**Authors:** 


### SPECIAL ARTICLES.

### CONSUMPTION AND DOGMA.

It is a good thing, no doubt, that there should exist capable people thoroughly in accord with the prevailing trend of opinion, always in the fashion with their ideas. A good thing, that is, not only for themselves (for, of course, theirs is the happy lot of the man who " shouts with the biggest crowd "), but in order that some sort of approximation to the truth may get itself well formulated, and attract needful attention. In a little book* just published, Dr. Latham gives further proof that professionally he is just this sort of person. The book is called an " economic study," and is the product of joint authorship; but necessarily a great deal of purely medical interest has to precede the ancillary question of pounds, shillings, and pence, and, moreover, this is a medical journal. Our remarks will therefore be confined to what Dr. Latham has to say.

Of much of it we heartily approve. He shows clearly at what an absurdly small relative cost anti-tuberculosis dispensaries on the Continental or Scotch systems could be established in England, and what a practical improvement on existing out-patient departments they would constitute. His insistence that the measures now adopted are merely directed towards alleviation of distress in the present generation, though there is nothing very new about it, is absolutely just. He has hit the truth in his statement that personality and devotion in the sanatorium medical superintendent are essential, and that to the right sort of man a free hand should be given. There is a rather pat aphorism that citizens have a right to be protected against an evil product of civilisation.

His style of writing, too, if not quite that of a literary man, or as beautifully clear as Mr. Lockwood's or Dr. Green's clinical lectures, has a certain " classical " quality which makes it good reading. It is, however, no exaggeration to say that the lay reader (and the book plainly caters for lay readers) will get the impression (especially as the laity always takes its medical man literally) that consumption is nowadays always preventable and always curable. Indeed, the following passages can mean hardly anything else:—P. 13 . . . " it can be prevented, absolutely, by intelligent effort and co-operation in the course of a comparatively short period of time." P. 129, " The provision of curative treatment is a question of cost. If a consumptive is well supplied with money, and is capable of following wise advice, he may even now feel sure that there is little to fear as to his future." It is confidently stated that in the sanatorium patient " relapse, or an extension of the disease, is always to be traced to excessive exertion having brought about the absorption of an excessive dose of poison." Responsible people may be led to believe that a man can only get tuberculosis if " his defensive forces are run down " (rather a " derangement of epitaphs," by the way) from drink, privation, insanity, or previous illness.

Now, it is almost a pity that this view of the question is not correct, because it is so delightfully simple and promising. Actual matters of fact have a way of being far more complex, and causation as found in nature, even so far as present knowledge goes, nearly always involves a plexus of factors. Like others, Dr. Latham borrows Professor Osler's illustration of tuberculous predisposition by the Biblical parable of the sower; but he leaves out, as Professor Osler is too wise to do, the factor of *intrinsic* predisposition. If, said Sir William Broadbent, good environment counts for everything, one should hardly ever see tubercle in the upper classes; but, he added, it does get there somehow. Dr. Mott, whose sympathy with pathological views will hardly be questioned, has boldly stated that his observation of the disease in lunatics disposes him

* "The Conquest of Consumption: an economic study." By A. Latham, M.D., and Chas. H. Garland. (London: T. Fisher Unwin. 1910.)

to think it may have a rather beneficial selective effect on the race. It must not be forgotten that no published work exists directly disproving the existence, surmised since earliest times, of a special tuberculous diathesis; and at the present day Beneke's careful researches, and anatomical observations like those we noticed lately of Professor Geddes, and references to mouth-breathing as a contributory factor in several recent papers on phthisis, including this year's Parkes-Weber prize essay, all point in this direction. There is quite a little literature, for instance, on the frequency of polymastia and supernumerary nipples in the consumptive. Those, then, who wish to prove the negative that the consumptive differs originally and intrinsically in no way from anybody else must first meet and dispose of all such obvious objections. Then as to Dr. Latham's enthusiastic contentions that the rich patient who will do as a good chest specialist tells him is certain to recover, and that no inexplicable relapses occur of patients under treatment. Well, these things may be, but one asks, after the example already quoted, why obituary notices of consumptives in easy circumstances are still common, and what point there is in the remarkably frank declaration of an eminent French clinician, to the effect that the only thing certain in the prognosis of pulmonary tuberculosis is that the prophet will be wrong.

In fine, Dr. Latham's orthodoxy is extreme, his book somewhat dogmatic. Yet, as already hinted, this is not altogether to be regretted. In matters of public health, spite of the good record of this country, harm has never yet come of too rapid progress; it is hard to over-urge the authorities. But this, perhaps, may be said. Deductions, mainly founded on bacteriology, have proved wrong over consumption already in one notable instance, and the laity has not forgotten it. If performance fall short of promise again, medical prestige will suffer.

